# Looking Māori Predicts Decreased Rates of Home Ownership: Institutional Racism in Housing Based on Perceived Appearance

**DOI:** 10.1371/journal.pone.0118540

**Published:** 2015-03-04

**Authors:** Carla A. Houkamau, Chris G. Sibley

**Affiliations:** The University of Auckland, Auckland, New Zealand; Mälardalen University, SWEDEN

## Abstract

This study examined differences in rates of home ownership among Māori (the indigenous peoples of New Zealand). We identified systematic factors that predicted why some Māori were more likely to own their own home (partially or fully) relative to other Māori. Data were drawn from a large national postal sample of 561 self-identified Māori collected as part of the New Zealand Attitudes and Values Study. As predicted, our analyses indicated that self-reported appearance as Māori, or the extent to which people thought they personally displayed features which visibly identified them as Māori to others, significantly predicted decreased rates of home ownership. This association held when adjusting for numerous demographic covariates, such as education, level of deprivation of the immediate area, household income, age, relationship status, region of residence, and so forth. Our analyses suggest there is, or at least has been in the recent past, institutional racism against Māori in New Zealand’s home lending industry based on *merely appearing more Māori*.

## Introduction

Perceived Stereotypicality (PS) is defined as “the degree to which a group is viewed in a stereotypic fashion, that is, as possessing stereotypic attributes to a large degree and as not possessing counter-stereotypic attributes” (p.461) [1]. A growing body of international research demonstrates that the more members of minority groups possess attributes considered stereotypic of their group, the more vulnerable they are to discriminatory treatment [2]. For example, research in the United States has shown that the likelihood of being sentenced to death for African American defendants is influenced by the degree to which they are perceived to have a stereotypically Black appearance [3]. According to Eberhardt and colleagues, looking ‘death-worthy’ for African Americans depends, at least in part, on the darkness of their skin.

Thankfully New Zealand, where the current study was conducted, does not have the death penalty. However, there are other ways in which perceived stereotypicality may powerfully affect how ethnic minorities are evaluated and treated. In this paper, we test whether perceived stereotypicality is linked to institutional racism against Māori in relation to home ownership.

Māori are the indigenous peoples of New Zealand. They are the second-largest ethnic group in New Zealand, after European New Zealanders. According to the 2013 New Zealand Census roughly 15% of the New Zealand population identify their ethnicity as Māori [4]. Understanding Māori social and economic challenges have practical implications in New Zealand as Māori continue to feature prominently in many negative social statistics [5].

With regard to housing and home ownership, for instance, researchers have noted that according to the 2006 New Zealand Census, Māori were four times more likely than Europeans to live in overcrowded, poor quality homes and more likely to be dependent on state provided housing [6, 7]. Here, we present a statistical model assessing whether there are reliable differences in rates of home ownership *within* the Māori population, that is, the extent to which some Māori are more likely to own their own home relative to other Māori. Specifically, we test whether Māori who perceive themselves as appearing more stereotypically Māori are less likely to own their own home relative to those who believe they appear less stereotypically Māori. We argue that merely looking more stereotypically Māori decreases the likelihood of having a mortgage application approved by a bank or financial institution—or, in other words, cause one to look less ‘mortgage-worthy.’ If this is the case, then we should find a reliable association between subjective reports of one’s appearance as Māori and decreased rates of home ownership within this population.

### Home Ownership in New Zealand

Owning one’s own home is associated with an advantaged socioeconomic position as well as other, more subjective elements of personal security and wellbeing. According to 2013 national Census data, just under half (49.8%) of New Zealanders aged 15 years and over own their home, compared with 53.2% in 2006 [8, 9]. Home ownership, it seems, is on a general decline in New Zealand.

However, as with many indicators of wellbeing, there were substantial differences in home ownership between ethnic groups. Europeans were the most likely to be home owners, with 56.8% owning their own home, a rate slightly higher than the national average. Home ownership for each of the other main ethnic groups was: Asian—34.8%, Māori—28.2% and Pacific peoples—18.5% [9].

Part of this dramatic ethnic disparity in the rate of home ownership may be due to differences in other ‘third’ variables. For example, there are differences in ownership rates across regions, with home ownership being lowest in Auckland (47.5%), and Pacific people also being more likely to reside in Auckland [9]. Similarly, part of the difference across groups may be due to differences in the average age of the population. Māori tend to die younger than Europeans, for example, and census data indicate that older people are more likely to own their own home. Nevertheless, these demographic differences in age and location fail to fully account for the gap between ethnic groups in relation to home ownership.

### Appearance and discrimination

There is now a strong body of evidence documenting differences in experiences of institutional racism depending on one’s stereotypical appearance as a member of a disadvantaged ethnic/racial minority. The majority of this research has focused on the outcomes experienced by African Americans in the North American context [3, 10–13].

In New Zealand, inequality is also evident throughout the justice system [14]. Māori are approximately 3.5 times more likely to be apprehended for a criminal offence than non-Māori. According to Workman, the level of Māori over-representation in the criminal justice system has reached a level similar to African Americans in the US [15]. Workman observed that while other factors contributing to Māori criminal offending (such as poverty and unemployment) are openly acknowledged, no response or acknowledgment is made of systematic bias within the New Zealand criminal justice system.

The disparity in outcomes experienced by Māori relative to Europeans in New Zealand has been demonstrated in relation to health and education. For example, data from the 2006/07 New Zealand Health Survey shows that Māori who tended to be viewed as European by others (and thus presumably were low in perceived stereotypicality) reported significantly lower rates of racial discrimination relative to those who looked more stereotypically Māori [16]. Turner’s study of the attitudes of New Zealand teachers towards students of different ethnic groups also suggested that there may be a bias against Māori pupils [17]. Turner collected qualitative data and argued that their interviews indicated that teachers tended to have lower expectations for, and more negative beliefs about, Māori students than any other ethnic group represented in New Zealand classrooms.

Against this backdrop, we expect a similar bias in the rates of home ownership experienced by Māori who are high in perceived stereotypicality relative to those who look less Māori. We should be able to detect the cumulative effect of this bias by testing whether Māori who rate themselves as more subjectively Māori looking are less likely to own their own home. If present, this effect would most likely reflect a systematic bias in lending by banking institutions that has been present over a long period of time, with banks being less likely to offer home loans to Māori people who, simply put, look more Māori. This systematic bias, would have, in effect, ‘built up’ over time into a cumulative difference in the population. We can detect this statistically by assessing whether Māori who are more visibly ethnically Māori, are less likely, on average and adjusting for other factors, to own their own home at the present date (i.e., when our sample was collected in 2013). It may be that this bias does not exist today, but if detected our analysis would suggest that it has certainly existed in the recent past.

Of course, there are numerous other factors that might also account for an association between variation in perceived stereotypically and home ownership amongst Māori. Perhaps people who look more Māori earn less (which would arguably be itself a form of institutional racism), or perhaps there are differences across regions (urban versus rural) that coincide with home ownership rates and also Māori appearance. Our statistical analysis aims to adjust for, and thus rule out, numerous such potential confounding demographic factors.

To the extent that we have been successful in identifying and adjusting for such factors we assert that any remaining statistical ‘difference in outcomes’ experienced by Māori who look more Māori is a form of institutional racism.

### Measuring Perceived Appearance

Research on stereotypicality and racial discrimination in the North American context has tended to use objective ratings of people’s faces and then look for variation in the outcomes based on these ratings. One common procedure for this type of research is to have independent observers rate the faces of members of the disadvantaged or minority group in terms of their perceived stereotypicality. This is the procedure that Eberhardt and colleagues used in their ‘looking deathworthy’ study [3]. However, it is possible to simply ask people to rate the extent to which they themselves think they look like a representative member of their ethnic group. This is the approach we use in the current research.

Houkamau and Sibley recently updated their Multidimensional Model of Māori Identity and Cultural Engagement (MMM-ICE) to include a subscale designed explicitly to measure perceived Māori appearance [18]. The Perceived Appearance subscale is defined as assessing the extent to which the individual subjectively evaluates their appearance as having clear and visible features that signal their ethnicity and ancestry as Māori (or high Māori prototypicality) *versus* the extent to which the individual evaluates their appearance as less indicative of having Māori ancestry (low Māori prototypicality). Their measure of Perceived Appearance was designed and validated explicitly for use within a Māori research context. The scale asks people to rate their level of agreement or disagreement with items such as ‘I think it is easy to tell that I am Māori just by looking at me’ and the reverse worded items such as ‘people would never know that I am of Māori descent just by looking at me.’

Houkamau and Sibley validated their measure of Perceived Appearance by showing that it predicted concurrent levels of perceived discrimination [18]. That is, as would be expected based on stereotypicality effects in other nations, they showed that Māori who reported that they looked more Māori also reported higher levels of racial discrimination. Their validation study did not, however, explore which types of discrimination Māori may experience on the basis of Perceived Appearance. Rather, their research simply examined variation in ratings of the subjective statement ‘I feel that I am often discriminated against because of my ethnicity.’ These findings—the correlation between subjective ratings of one’s appearance and perceived discrimination—held when statistically adjusting for numerous other aspects of identity, such as how socio-politically engaged one was, and general ratings of how important one felt being Māori was to one’s self-concept.

### Overview of the present study

In this study, we test a statistical model assessing the factors that predict home ownership among Māori. We test this model using data from a large national postal sample of Māori. The data are analysed using logistic regression analysis, in which the likelihood of being a full or partial home owner (coded as 1) versus not being a home owner (coded as 0) was predicted using a broad range of demographic factors and subjective elements of Māori identity. Our data are based on a self-report questionnaire, and thus our outcome is self-reported home ownership to a yes/no question: ‘Do you own your own home (either partially or fully)?’

We first present a baseline demographic model, which assesses the extent to which, for Māori, the likelihood of owning one’s own home is predicted by gender, age, household income, level of education, employment, religious status, parental status, relationship status, residential status (urban versus rural), the deprivation of each participants’ immediate neighbourhood, and whether or not participants lived in the Auckland region versus other regions of the country. Our aim here was to include as broad a range of demographic factors as possible, and to thus account for the covariance of all of these demographics in our model. We then extended our analysis to also include scores on each of the seven dimensions of Māori identity identified by Houkamau and Sibley in their MMM-ICE2 [18].

Our model thus allowed us to first examine the extent to which different demographic factors uniquely predicted home ownership when adjusting for other demographics. By including Māori identity scale scores at the second step, our model then allowed us to determine the extent to which different aspects of one’s subjective experience as Māori predicted home ownership when adjusting for demographics, and also when adjusting for scores on other aspects of Māori identity. This is an important point, because our approach thus allowed us to test whether subjective ratings of one’s appearance as Māori (how visibly Māori one sees oneself as being) predicted the likelihood of home ownership in a way that could not be explained or accounted for by either demographic factors, or other aspects of Māori identity.

Stated formally, we hypothesised that: Māori who scored higher on the Perceived Appearance subscale of the MMM-ICE2 would be less likely to own their own home (either partially or fully), relative to Māori who scored lower on this subscale, and thus rated themselves as subjectively less visibly identifiable as Māori. This effect should hold (remain significant) when adjusting for numerous demographic covariates, as well as other aspects of subjective identification as Māori.

## Method

### Participants

This study was approved by the University of Auckland Human Participants Ethics Committee on 17-February-2012 until 09-September-2015. Reference Number: 6171. Participants were 561 Māori (354 women, 207 men) who completed the New Zealand Attitudes and Values Study (NZAVS) Time 4 Māori Focus questionnaire, identified their ethnicity as Māori (either solely, or along with other ethnic affiliations), and who provided complete responses to all of the measures analysed here (except 105 missing values for income, which were replaced with the sample mean in our regression model). Participants had a mean household income of $77,880 (SD = $71,630).

Participants had a mean age of 45.06 years (SD = 12.53, range 18–69). With regard to other demographics, 46.9% were religious, 87.2% were parents, 59% were in a committed romantic relationship, 66% were employed, and 52% lived in an urban area (as opposed to a rural area of New Zealand). Participants’ locations were also coded with regard to whether they lived in the Auckland region specifically, and 28.5% did. Auckland is the largest city in New Zealand, and property prices in Auckland tend to be far higher than other regions of the country. Education was coded as an ordinal variable with the categories: none/unreported (23.5%), a high school qualification (33.3%), a diploma or certificate (18.7%), an undergraduate degree, or studying toward one (16.9%) a post-graduate degree or studying toward one (7.5%).

### Sampling procedure

The Time 4 NZAVS sampled a total of 12,183 participants. As part of the sampling design, this phase of the NZAVS included a booster sample aimed specifically at recruiting Māori participants (Frame 5 of the Time 4 NZAVS). This sample frame consisted of 9,000 people randomly selected from those who indicated on the 2012 Electoral Roll that they were of Māori descent (Māori descent is listed on the role, but other ancestry information is not). A total of 690 participants responded to this booster sample. When completing the questionnaire, 670 respondents identified their ethnicity as Māori, and 20 did not. We limited our analyses to participants who ethnically identified as Māori, as this is an assumed pre-condition for completing the MMM-ICE subjective measure of Māori identity.

At the time of sampling, the electoral roll had a stated accuracy of 98.5%. When adjusting for the overall address accuracy of the electoral roll, this represents an (adjusted) response rate of 7.78%. It should be noted that this response rate is lower than that observed for the main (full random probability) sample frames used in the NZAVS, which give responses rates of up to approximately 16%. The low response rate for this sample likely indicates a combination of factors, among the most influential being the overall reduced likelihood of Māori participants to respond to postal surveys in general, combined with the possibility that contact details for Māori in the electoral roll may, on average, have a lower level of accuracy [19]. It is likely that this relatively low response rate was also partially affected by the fact that people were opting into a 15-year longitudinal study, and providing their contact details indicated that they were willing to be contacted by us to complete similar questionnaires for the next 15 years.

The questionnaire administered to the NZAVS Māori booster sample included questions specifically designed for Māori, and the cover letter introduced the survey as a ‘The New Zealand Attitudes and Values Study—Māori Identity Focus Questionnaire.’ The lead researcher and primary point of contact for this sample frame was of Māori descent, and was introduced to participants in the cover letter by listing Iwi affiliations. Participants were informed that they had been randomly sampled for this study from among those who indicated that they were of Māori descent on the electoral roll. The questionnaire was similar in format and content to the standard NZAVS questionnaire, with the exception that it included approximately 2 pages of questions designed specifically to assess aspects of identity and wellbeing specifically for Māori, and in Māori cultural contexts.

### Small-area deprivation

We measured the deprivation of participants’ immediate (small area) neighborhood using the 2013 New Zealand Deprivation Index [20]. New Zealand is unusual in having rich census information about each area unit/neighborhood of the country available for research purposes. The smallest of these area units are meshblocks. Statistics New Zealand defined a meshblock as “a defined geographic area, varying in size from part of a city block to large areas of rural land” [21]. Each meshblock abuts against another to form a network covering all of New Zealand including coasts and inlets, and extending out to the two hundred mile economic zone. The geographical size of these meshblock units differs depending on population density, but each unit tends to cover a region containing a median of roughly 90 residents (M = 103, SD = 72, range = 3–1,431).

The New Zealand Deprivation Index uses aggregate census information about the residents of each meshblock to assign a decile-rank index from 1 (least deprived) to 10 (most deprived) to each meshblock unit [20]. Because it is a decile-ranked index, the 10% of meshblocks that are most affluent are given a score of 1, the next 10% a score of 2, and so on. The index is based on a principal components analysis of the following nine variables (in weighted order): the proportion of adults who received a means-tested benefit, household income, proportion not owning their own home, proportion single-parent families, proportion unemployed, proportion lacking qualifications, proportion household crowding, proportion with no telephone access, and proportion with no car access.

The New Zealand Deprivation Index thus reflects the average level of deprivation for small neighborhood-type units (or small community areas) across the entire country. A key strength of the Deprivation Index is that because it is based on information about residential location, the measure does not rely on self-reports of socio-economic status, as survey measures of income or education do. The index is a well-validated index of the level of deprivation of small area units, and has been widely used in health and social policy research examining numerous health outcomes, including mortality, rates of hospitalization, smoking, cot death, and access to health care, to name just a few examples [22–25]. The index is also widely used in service planning by government and local council, and is a key indicator used to identify high needs areas and allocate resources such as health funding [26, 27]. The current sample had a mean deprivation index of 6.75 (SD = 2.75).

### Questionnaire measures

Home ownership was measured using the question ‘Do you own your own home (either partly or fully owned)?’ with a forced choice yes/no response option. This item was deliberately designed to be very general and non-specific, and thus aimed to identify anyone who owned either party or fully any type of dwelling which they considered their home.

Perceived stereotypically was measured using the Perceived Appearance subscale of the MMM-ICE2 [18]. This subscale contains the following seven items:

- ‘I think it is easy to tell that I am Māori just by looking at me.’
- ‘You only need to look at me to see that I am Māori.’
- ‘I think it is clear to other people when they look at me that I am of Māori descent.’
- ‘When people meet me, they often do not realize that I am Māori’ (reverse worded).
- ‘I think it is hard to tell that I am Māori just by looking at me’ (reverse worded).
- ‘People would never know that I am of Māori descent just by looking at me’ (reverse worded).
- ‘People who don’t know me often assume that I am from another (non-Māori) ethnic group’ (reverse worded).

As with all other items in the MMM-ICE, each item was rated on a scale from 1 (strongly disagree) to 7 (strongly agree). Reverse worded items were recoded and items were averaged to provide a mean subscale score, with higher values indicating higher levels of subjective perceived stereotypically (M = 4.34, SD = 1.82, α = .92, range 1–7). This subscale was validated by Houkamau and Sibley, and provides an internally reliable indicator of subjective prototypicality that is statistically independent of the six other aspects of Māori identity included in the MMM-ICE2 [18].

The other six factors of the MMM-ICE2 were also assessed. These were: Group Membership Evaluation (M = 5.73, SD = 1.10, α = .83), Cultural Efficacy and Active Identity Engagement (M = 4.78, SD = 1.37, α = .85), Spirituality (M = 5.24, SD = 1.42, α = .89), Socio-Political Consciousness (M = 5.08, SD = 1.44, α = .88), Authenticity Beliefs (M = 3.59, SD = 1.01, α = .61) and Interdependent Self-Concept (M = 5.78, SD = 1.35, α = .80). Construct definitions for each of these subscales are presented in Table 1. A full copy of the MMM-ICE is provided in Houkamau and Sibley [5], and the revised version and additional information about scale validity is presented in Houkamau and Sibley [18] and Sibley and Houkamau [28].

**Table 1:** Construct definitions for the seven factors indexed by the MMM-ICE2 [18]

***Group Membership Evaluation (GME)***: The extent to which the individual positively evaluates their membership in the social category Māori and views their membership as Māori as a personally important or central aspect of their self-concept versus the extent to which the individual negatively evaluates their membership in the social category Māori and views their membership as Māori as peripheral or irrelevant to their self-concept.
***Cultural Efficacy and Active Identity Engagement (CEAIE)***: The extent to which the individual perceives that they have the personal resources required (i.e., the personal efficacy) to engage appropriately with other Māori in Māori social and cultural contexts versus the extent to which the individual perceives that they lack the personal resources and ability to engage appropriately with other Māori in Māori social and cultural contexts.
***Interdependent Self-Concept (ISC)***: The extent to which the concept of the self-as-Māori is defined by virtue of relationships with other Māori people versus the extent to which the concept of the self-as-Māori is viewed as being defined as solely unique and independent to the individual rather than as part of the social group.
***Spirituality (S)***: The extent to which the individual is engaged with, and has a belief in, certain Māori concepts of spirituality, including a strong connection with ancestors, Māori traditions, the sensation and experience of *waahi tapu* (sacred places), and a strong spiritual attachment and feeling of connectedness with the land versus the extent to which the individual is disengaged from or does not believe in Māori concepts of spirituality.
***Socio-Political Consciousness (SPC)***: The extent to which the individual perceives historical factors as being of continued importance for understanding contemporary intergroup relations between Māori and other ethnic groups in New Zealand; and how actively engaged the individual is in promoting and defending Māori rights given the context of the Treaty of Waitangi versus the extent to which the individual perceives historical factors and injustices experienced by Māori as being irrelevant in contemporary society.
***Authenticity Beliefs (AB)***: The extent to which the individual believes that to be a ‘real’ or ‘authentic’ member of the social category Māori one must display specific (stereotypical) features, knowledge and behaviour versus the extent to which the individual believes that Māori identity is fluid rather than fixed, and produced through lived experience.
***Perceived Appearance (PA)***: The extent to which people subjectively evaluate their appearance as having clear and visible features that signalling their ethnicity and ancestry as Māori (or high Māori prototypicality) versus the extent to which people evaluate their appearance as less indicative of having Māori ancestry (low Māori prototypicality).

### Overview of data analysis

A logistic regression model was conducted predicting likelihood of owning (partially or fully) one’s own home (1 = yes, 0 = no). We estimated the model using Maximum Likelihood with robust estimation of the standard errors. This is a conservative approach which adjusted for possible non-normality in the distribution of residuals (note that comparable results were observed under simple Maximum Likelihood). We adopted a p <. 01 as our criteria for statistical significance. This is a relatively conservative threshold for determining statistical significance (p <. 05 is more typical). However, we wanted to err on the side of caution and further reduce the risk of detecting spurious effects.

We first tested a demographic model including a broad range of demographic variables that might potentially explain variation in home ownership for Māori. Our goal here was to include as broad a set of demographic covariates as possible, and thus rule out the possibility that the predicted association between Perceived Appearance and home ownership might be due to some alternative demographic factor. The demographic variables we included were: gender, age, household income, level of education, employment, religious status, parental status, relationship status, residential status (urban versus rural), the deprivation of each participants’ immediate neighbourhood, and whether or not participants lived in the Auckland region versus other regions of the country.

We then extended this model to include scores on each of the seven dimensions of Māori identity: Group Membership Evaluation, Cultural Efficacy and Active Identity Engagement, Interdependent Self-Concept, Spirituality, Socio-Political Consciousness, Authenticity Beliefs, and Perceived Appearance.

This approach allowed us to determine the extent to which simple demographic factors predicted the whether specific aspects of subjective identification as Māori uniquely predicted home ownership when statistically adjusting for alternative demographic or regional factors that might also explain the effect.

Demographic characteristics and mean subscale scores for home owners and non-owners are presented in Table 2.

**Table 2:** Descriptive statistics and summary sample information for self-identified Māori by home non-owners versus owners.

	Non-Home Owner		Home Owner	
	%	*N*	%	*N*
Gender Men	44.0%	91	56.0%	116
Women	51.1%	181	48.9%	173
Education;None	70.5%	93	29.5%	39
High School	49.7%	93	50.3%	94
Trade Cert	36.2%	38	63.8%	67
Undergraduate	38.9%	37	61.1%	58
Post-grad	26.2%	11	73.8%	31
Employment Employed	40.3%	149	59.7%	221
Unemployed	64.4%	123	35.6%	68
Religion: Religious	52.5%	138	47.5%	125
Non-religious	45.0%	134	55.0%	164
Parent:Parent	75.0%	54	25.0%	18
Non-parent	44.6%	218	55.4%	271
Relationship:Partnered	31.4%	104	68.6%	227
Single	73.0%	168	27.0%	62
Urban: Rural	43.9%	118	56.1%	151
Urban	52.7%	154	47.3%	138
Auckland:Auckland	53.1%	85	46.9%	75
Non-Auckland	46.6%	187	53.4%	214
	*M*	*SD*	*M*	*SD*
Age (years)	42.20	13.22	47.75	11.22
Household income (NZ$)	$50,516	$35,811	$99,643	$96,254
NZ Deprivation Index	7.39	2.51	6.15	2.83
Group Membership Evaluation	5.84	1.07	5.64	1.11
Cultural Efficacy	4.90	1.39	4.66	1.35
Interdependent Self-Concept	3.95	1.43	3.63	1.26
Spirituality	5.31	1.42	5.18	1.42
Socio-Political Consciousness	5.16	1.36	5.00	1.51
Authenticity Beliefs	3.73	1.03	3.45	0.96
Perceived Appearance	4.61	1.78	4.09	1.82

Note that estimates for income did not include the 105 cases replaced with the sample mean.

### Results

The demographic model predicting the likelihood of owning (partially or fully) one’s own home explained 49.2% of the variance (R^2^ = .492, se = .050, z = 9.783, p <. 001). The full demographic-identity model predicted a grand total of 53.5% (R^2^ = .535 se = .029, z = 3.210, p < .001).

Perceived Appearance predicted a significant decrease in the likelihood of owning one’s own home when examined in isolation without any other covariates in the model (b = -.161, se = .043, unadjusted OR = .851, 95% CI of unadjusted OR = [.793, .914], t = -3.734, p <. 001). Critically, and as reported in Table 3, the link between Perceived Appearance and reduced rates of home ownership remained significant when adjusting for the full set of demographic covariates, and other aspects of Māori identity and cultural engagement (b = -.199, se = .070, adjusted OR = .820, 95% CI of adjusted OR = [.715, .940], t = -2.837, p = .005).

**Table 3:** Regression slopes and odds ratios for the model predicting likelihood of owning (partially or fully) one’s own home for Māori participants.

	Logistic regression model predicting likelihood of owning one’s own home (either partially or fully) relative to not owning a home.					
	B	se	OR	95% CI of OR	t	p
**Demographic model**						
Intercept/Threshold	3.624	.638				
Gender (0 female, 1 male)	.071	.219	1.074	[0.700, 1.648]	.326	.745
Age (years)	.048	.009	1.049	[1.030, 1.069]	5.096*	<.001
Income ($10,000 units)	.134	.029	1.144	[1.080, 1.212]	4.577*	<.001
Education (ordinal-2 to 2)	.348	.091	1.416	[1.185, 1.692]	3.834*	<.001
Employment (0 no, 1 yes)	.471	.229	1.601	[1.023, 2.507]	2.060	.039
Religion (0 no, 1 yes)	-.606	.215	.545	[0.358, 0.831]	-2.822*	.005
Parent (0 no, 1 yes)	.725	.376	2.065	[0.988, 4.316]	1.928	.054
Relationship (0 no, 1 yes)	1.202	.218	3.326	[2.169, 5.101]	5.509*	<.001
Urban (0 rural, 1 urban)	-.512	.231	.599	[0.381, 0.943]	-2.212	.027
NZ Deprivation Index 2013 (1–10)	-.055	.043	.946	[0.870, 1.029]	-1.299	.194
Auckland Region (0 no, 1 yes)	-.009	.258	.991	[0.598, 1.643]	-.035	.972
**Demographic-Identity model**						
Intercept/Threshold	2.063	.843				
Gender (0 female, 1 male)	.168	.233	1.183	[0.750, 1.867]	.723	.470
Age (years)	.051	.010	1.052	[1.032, 1.072]	5.239*	<.001
Income ($10,000 units)	.140	.031	1.150	[1.081, 1.223]	4.440*	<.001
Education (ordinal-2 to 2)	.343	.098	1.409	[1.163, 1.706]	3.501*	<.001
Employment (0 no, 1 yes)	.490	.234	1.632	[1.032, 2.581]	2.096	.036
Religion (0 no, 1 yes)	-.543	.226	.581	[0.373, 0.906]	-2.397	.017
Parent (0 no, 1 yes)	.936	.407	2.549	[1.148, 5.663]	2.298	.022
Relationship (0 no, 1 yes)	1.199	.225	3.317	[2.133, 5.157]	5.324*	<.001
Urban (0 rural, 1 urban)	-.547	.237	.579	[0.364, 0.921]	-2.308	.021
NZ Deprivation Index 2013 (1–10)	-.028	.045	.972	[0.890, 1.061]	-.633	.527
Auckland Region (0 no, 1 yes)	-.061	.260	.941	[0.565, 1.567]	-.234	.815
Group Membership Evaluation	-.247	.150	.781	[0.582, 1.048]	-1.650	.099
Cultural Efficacy	.015	.117	1.015	[0.806, 1.278]	.128	.898
Interdependent Self-Concept	-.080	.099	.923	[0.761, 1.120]	-.813	.416
Spirituality	.191	.117	1.211	[0.962, 1.523]	1.632	.103
Socio-Political Consciousness	.034	.103	1.035	[0.845, 1.267]	.331	.740
Authenticity Beliefs	-.222	.114	.801	[0.641, 1.002]	-1.943	.052
Perceived Appearance	-.199	.070	.820	[0.715, 0.940]	-2.837*	.005

Notes. 0 = not owning one’s own home, 1 = owning (partially or fully) one’s own home. Fit indices for demographic model: *R* ^*2*^ = .492, *se* = .050, *z* = 9.783, *p* <. 001. Fit indices for demographic-identity model: *R* ^*2*^ = .534 *se* = .049, *z* = 10.828, *p* <. 001. OR = Odds Ratio,

* p <. 01. n = 561.

The Odds Ratio of. 820 in our model with covariates included indicates that for each one-unit increase in ratings of Perceived Appearance (keeping in mind that scale scores ranged from 1–7), Māori were. 82 times less likely to own their own home. Put another way, what our results suggest is that for every one-unit decrease in one’s subjective appearance as Māori, the odds of either owning one’s own home (in part or in full) went up by 1.22.

Finally, and unsurprisingly, the results reported in Table 3 indicate that Māori who were older were more likely to own their own home. Māori with a higher household income, who were more educated, and in a committed relationship were also more likely to own their own home. However, these results did not account for the effect of Perceived Appearance.

## Discussion

Census data clearly indicate that Māori are, on average, less likely than Europeans to own their own home. Broad differences do not tell us whether this difference in outcomes is experienced by all Māori equally, or whether there are *systematic differences in outcomes within the Māori population*. This study focused on differences in rates of home ownership among Māori and aimed to identify systematic factors that predicted why some Māori were more likely to own their own home, either partially or fully, relative to other Māori. More specifically, this study tested the hypothesis that differences in self-reported appearance as Māori, or the extent to which Māori people perceived their features identified them as stereotypically Māori to others, significantly predicted decreased rates of home ownership. It did.

Moreover, this association held when adjusting for numerous other possible demographic factors that might account for the association, such as education, deprivation of the immediate area, household income, age, relationship status, and so forth. The effect also held when statistically adjusting for other aspects of one’s subjective identification as Māori, such as levels of cultural efficacy and the level of one’s spiritual connection to the land.

Subjective ratings of Perceived Appearance were measured by asking Māori to rate how strongly they disagreed or agreed with statements such as ‘I think it is clear to other people when they look at me that I am of Māori descent’ and the reverse-worded statement ‘When people meet me, they often do not realize that I am Māori.’ These items were rated from 1 to 7 (a standard range for such scales). Our statistical model indicated that for every additional one-unit increase in how much people felt they looked Māori (on a range where 1 is the lowest score and 7 the highest), Māori were. 82 times less likely to own their own home. To think of this same effect in reverse, what our results suggest is that for every one-unit decrease in one’s subjective appearance as Māori, the odds of partly or fully owning one’s own home went up by 1.22.

Some readers may be wondering how large this effect is in practical terms. One way to think about it is like this: when statistically adjusting for numerous other demographics, such as differences in income, region of residence, and education, a Māori person with a score of 5.55 on our Perceived Appearance measure of Māori identity would be *twice as likely to not own their home* relative to someone with a score of 1 in Perceived Appearance. This is a statistically significant association, which in our view represents a large and extremely important difference in the rate of home ownership based solely on *merely appearing more Māori*.

To address a possible misconception: our analyses do not test whether there is a current bias in the likelihood of approving home loans to people who look more Māori. Indeed it is possible that this bias may not exist in present day home lending practises (this is an important question for future research). What our analyses are picking up on is a statistical signal that is most likely to have been produced by a systematic bias in lending by banking institutions over a fairly long period of time. This systematic bias has likely ‘built up’ over some undefined period of time to produce the cumulative difference in the proportion of Māori of various levels of perceived stereotypically who now own their own homes in New Zealand.

In the absence of other demographic or social factors which may explain the strong and robust link between looking more Māori and the decreased likelihood of home ownership, it is our considered opinion that the difference in home ownership rates among Māori on the basis of mere appearance constitutes an extremely important form of institutional racism. And one that likely has far-reaching implications for numerous ‘trickle-down’ aspects of the health and wellbeing of Māori as a result.

In many ways our data does not come as a surprise. Racism against Māori in New Zealand is commonplace throughout New Zealand institutions. In relation to housing the roots are historical. For example, Bierre et al.’s analysis of 1930s and 1940s New Zealand housing policies demonstrated how housing programmes have historically excluded Māori from administration, planning and decision making in relation to their own housing provisions [29]. Apart from institutional racism, research on discrimination in the private rental market, has revealed a range of negative stereotypes against Māori (such they are poor, risky, unclean tenants) which have not only justified covert discrimination but relegated Māori to poorer urban areas and cheap poor quality housing. For example, research carried out by MacDonald showed that barriers were put up to restrict the property available for Māori based on racial discrimination [30].

Our data aligns with previous studies which show racism and discrimination against Māori is widespread in New Zealand society and manifests in a wide variety of contexts. The incidence and impact of discrimination against Māori in various domains is well documented This includes health care, the media, the legal arena, and within New Zealand’s education system [15, 17, 31–35]. It is possible to interpret individual experiences of discrimination as just that; isolated incidents, and it is difficult to ‘prove’ that these isolated experiences reflect a broader and underlying level of institutional discrimination. By sampling a large number of individuals and looking for average trends, the current paper aimed to do precisely this. Our data tells us that these trends are not limited to the casual observations of a few individuals, but rather they show that the bias in home ownership for Māori who look more Māori is an institutional pattern. We can therefore conclude that the practices enacted within the banking industry prevent Māori with high perceived stereotypicality from accessing finance.

### Caveats and limitations

It is important to keep in mind that our measure of perceived stereotypicality is based on participants’ own subjective rating. Thus, people may be basing their judgements about how ‘Māori’ they look using multiple markers, including phenotypical features, Māori cultural tattoos and traditional adornments such as greenstone or bone carvings, their accent and whether one has a Māori name, etc. This measure has not been validated by matching people’s scores with independent ratings of their facial features. Our study is also cross-section in nature, thus we have not shown that perceived appearance predicts rates of change in home ownership. Rather, we detect a correlation between appearance and home ownership and show that this association remains when adjusting for numerous other ‘third variable’ demographics that might have also explained the association. Our analyses do not, however, adjust for intergenerational factors such as whether one’s parents owned their own home, or other factors associated with possible cycles of poverty. This is an important direction for future research.

### Broader implications and an agenda for future research

Our finding that higher perceived stereotypically relates to poorer outcomes for Māori contributes to international research that demonstrates that the way people look can have profound implications for their life experiences [3, 11–13]. Our findings also have several practical implications. Māori form a significant percentage of the New Zealand population and if Māori remain a marginalised underclass there is a social and economic cost to all New Zealanders [36–37]. At the same time, the Māori economy has grown significantly in recent decades and Māori are in a position to contribute to New Zealand’s economic future [38 – 40]. A key element of Māori economic development is the ability to access funding to grow their businesses and asset base. Our evidence suggests there is, or at least has been in the recent past, institutional racism against Māori in New Zealand’s home lending industry. This finding augurs poorly for Māori development overall. Future research may explore the implications of institutional racism against Māori in the financial services industry, implications for advancing Māori businesses, and indeed the New Zealand economy as a whole.

A comment on qualitative and quantitative research is also warranted. Many readers familiar with research on experiences of racism among Māori may be more familiar with qualitative or interview work. Some forms of institutional racism can be insidious and hard to detect when one focuses on a small number of individuals [41]. This is because you need to stand back to see these ‘hidden patterns’ that people may not be aware that they are part of. This type of racism is even more destructive for society as it is difficult to detect and reprimand. What our analyses aimed to do was detect these potentially ‘hidden’ patterns using statistical analyses of a large number of people.

### Concluding comments

This study focused on differences in rates of home ownership among Māori. The study aimed to identify systematic factors that predicted why some Māori were more likely to own their own home, either partially or fully, relative to other Māori. To sum up in one sentence: results from a large national probability sample of Māori indicate that the more Māori you look, the less ‘mortgage worthy’ you are.
